# Longitudinal surveillance of serum titanium ion levels in patients with indigenous 3D printed total temporomandibular joint replacement

**DOI:** 10.1038/s41598-023-33229-5

**Published:** 2023-05-04

**Authors:** Garima Khandelwal, Ajoy Roychoudhury, Ongkila Bhutia, A. Shariff

**Affiliations:** 1 Department of Oral and Maxillofacial Surgery, All India Institute of Medical Sciences, New Delhi, 110029 India; 2 Department of Anatomy, All India Institute of Medical Sciences, New Delhi, 110029 India

**Keywords:** Medical research, Materials science

## Abstract

The purpose of this longitudinal study was to surveil the serum titanium ion levels at various time intervals in patients with indigenous 3D-printed total temporomandibular joint replacement (TMJ TJR). The study was conducted on 11 patients (male: 8; female: 3) who had undergone unilateral or bilateral TMJ TJR. Blood samples were drawn preoperatively (T0), 3 months (T1), 6 months (T2), and 1 year (T3) postoperatively. Data were analyzed and a p value of < 0.05 was considered statistically significant. The mean serum titanium ion levels at T0, T1, T2, and T3 was 9.34 ± 8.70 µg/L (mcg/L), 35.97 ± 20.27 mcg/L, 31.68 ± 17.03 mcg/L, and 47.91 ± 15.47 mcg/L respectively. The mean serum titanium ion levels increased significantly at T1 (p = 0.009), T2 (p = 0.032), and T3 (p = 0.00) interval. There was no significant difference between unilateral and bilateral groups. Serum titanium ion continued to show increased levels till the last follow-up of 1 year. These initial serum titanium ion levels increase is due to the initial wear phase of the prosthesis which manifests over 1 year. Further studies with large sample sizes and long-term follow-ups are required to see the deleterious effect if any on the TMJ TJR.

## Introduction

Alloplastic total temporomandibular joint (TMJ) replacement (TJR) surgery has emerged as a worthwhile treatment modality in end-stage TMJ disease^1,2^. However, in recent years, much of the concern is being engrossed in the biocompatibility of metals used to reconstruct the joint and its leaching in the surrounding environment^3,4^. United States Food and drug association (USFDA) approved materials are used for the manufacturing of TMJ TJR which include ultrahigh molecular weight polyethylene (UHMWPE), chromium alloys (Co–Cr–Mo), alloyed titanium (Ti6Al4V), and commercially pure titanium (cpTi). But they do wear under functional loading and lead to the formation of metal debris around the prosthesis and surrounding soft tissues and may lead to a condition known as metallosis^4^. Metallosis is described as a medical condition that involves metal debris build-up and deposition in body tissues^5^. The entrapped metal debris between these surfaces of the prosthesis leads to integrated wear, thereby aggravating the condition^6,7^. This process of the buildup of metal debris due to mechanical friction has better described by the term tribocorrosion^7,8^. The study of friction, lubrication of material, and wear combined with the corrosion process is termed as tribocorrosion^7^.

**Figure 1:**
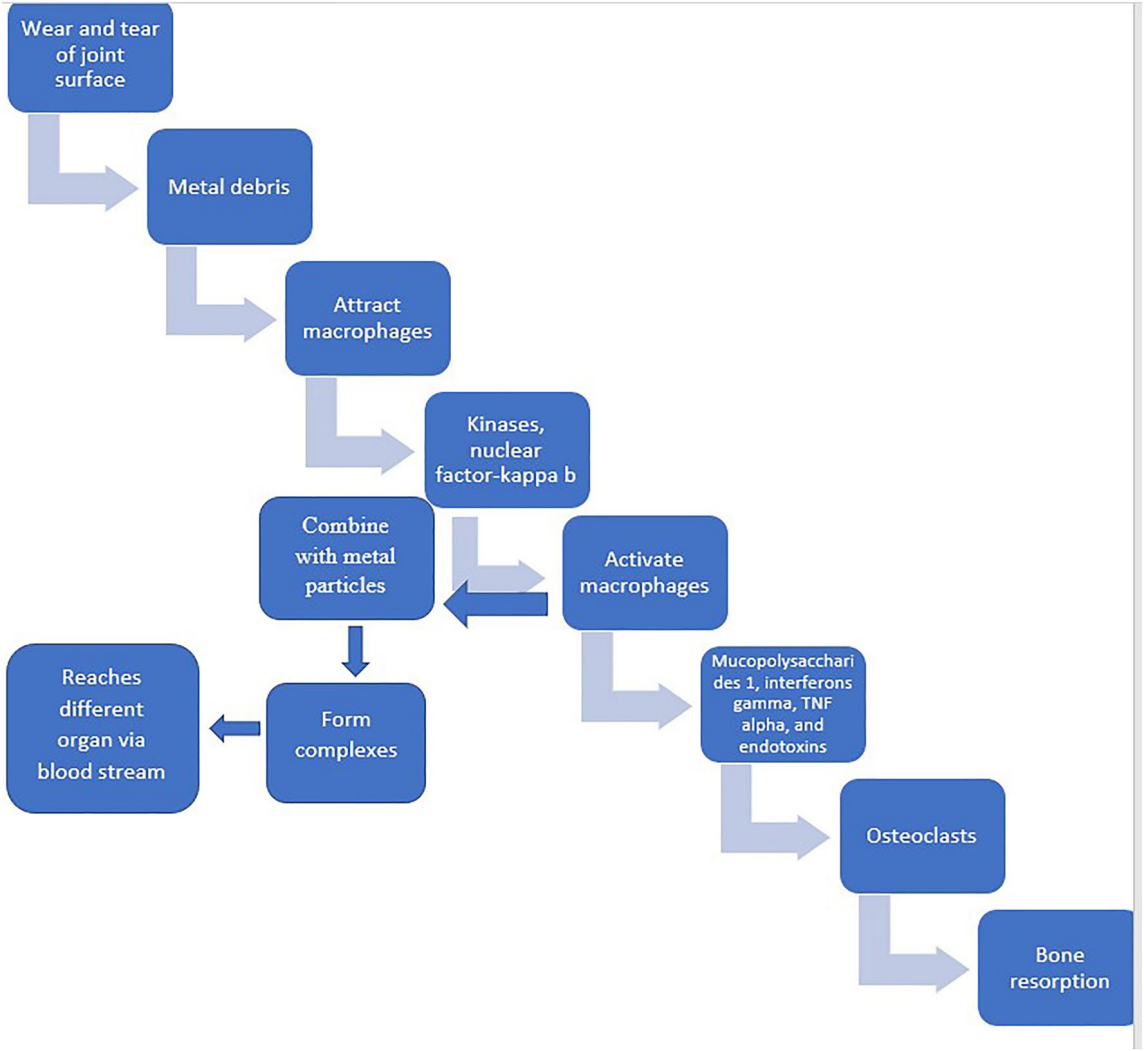
Pathogenesis of metallosis.

Wear and tear of the joint surface leads to the accumulation of metal debris around the joint surface. This debris polarizes macrophages, causing release of kinases and activation of nuclear factor-kappa b and activation of macrophages. These activated macrophages further secrete mucopolysaccharides 1, interferons gamma, TNF alpha, and endotoxins which conclusively lead to activation of osteoclasts that lead to bone resorption in the joint area. These macrophages combine with metal particles and form complexes that are further taken to different organs of the body via bloodstream leading to systemic symptoms and toxicity (Fig. 1)^9–11^.

There is no single parameter that can be used for the diagnosis of metallosis. To reach a final diagnosis, detailed history, thorough clinical examination of the patient combined with radiological findings and laboratory investigations including C reacting protein (CRP), erythrocyte sedimentation rate (ESR), lymphocyte transformation test (LTT), and serum, whole blood, and urine metal ion levels are required. The usual presenting signs and symptoms are pain, fever, skin discoloration around the prosthesis, crepitus, and tenderness in relation to the prosthesis. Other signs and symptoms may include variable presentation like combination of cardiovascular, neurological symptoms, and the endocrine symptoms which depends on the levels of the systemic metal ion. Radiological findings include the radiolucent area surrounding the prosthesis, dislodgement of the prosthesis which may indicate the osteolysis process. Correlation of all these signs and symptoms, radiological features, elevated CRP, ESR, LTT levels, and elevated blood, serum, and urine metal ions levels may help in diagnosing metallosis^12^.

Although the quality control is ensured during the manufacturing of the implants, the probability of metal leaching in surrounding tissues is not ruled out^13^. Chang et al. reported approximately 5.3% incidence of the metallosis after total hip arthroplasty^14^. Ollivere et al. reported a 3.1% incidence of metallosis after cognate prosthesis failure used in resurfacing of the hip^15^. There is abundant literature evidence in orthopedics regarding the serum metal ion levels and their consequences in total joint replacement, but there is dearth of literature in TMJ TJR patients^11,16^. This paucity of literature has created curiosity to design a study that will give an insight into the serum levels of titanium in TMJ TJR patients.

## Materials and methods

### Study design

This longitudinal study was initiated after getting approval from the “Institute ethical committee for post graduate research, All India Institute of Medical Sciences, New Delhi, India” (IECPG-698/19.12.2019) to evaluate the serum levels of titanium ions released from the TMJ TJR prosthesis. The study sample was composed of all the patients undergoing TMJ replacement surgery from January 2020 to December 2020 with age above 12 years. However, our centre mostly deals with TMJ TJR in patients having TMJ ankylosis, so in the present study, 11 patients which were included had ankylosis as the etiology. The methods were carried out in accordance with the institute protocol following the relevant guidelines and regulations. Written and informed consent was taken from all the patients participating in the study. The patients who had another implanted metallic device (dental implants, metallic crowns, knee joint replacement), having parafunctional habit or underlying systemic condition, or had a history of chronic usage of certain prescriptions like multivitamin supplements or working in a profession that leads to exposure to metal particles were excluded. However, 11 patients which were included in this study had long-standing ankylosis and nil jaw functions.

### Study variables

The primary objective was to assay serum concentration of titanium ions at preoperative (T0), 3 months (T1), 6 months (T2), and 1 year (T3) postoperatively. Secondary objectives were to compare serum titanium levels between unilateral and bilateral TMJ TJR, to observe and document the signs and symptoms of metallosis, if any.

### Sample size calculation

The sample size was calculated using n-Master Software. The known metallosis incidence after total hip replacement and total knee arthroplasty (Known population) is around 5 ± 2%. Considering the metallosis in TMJ TJR (Study group) to be less than total hip replacement as it is not high wear joint: it was kept at 3%. With Alpha error of 0.05 and 90% power of study; the sample size was calculated, n = 11.

### Type of joint

**Figure 2:**
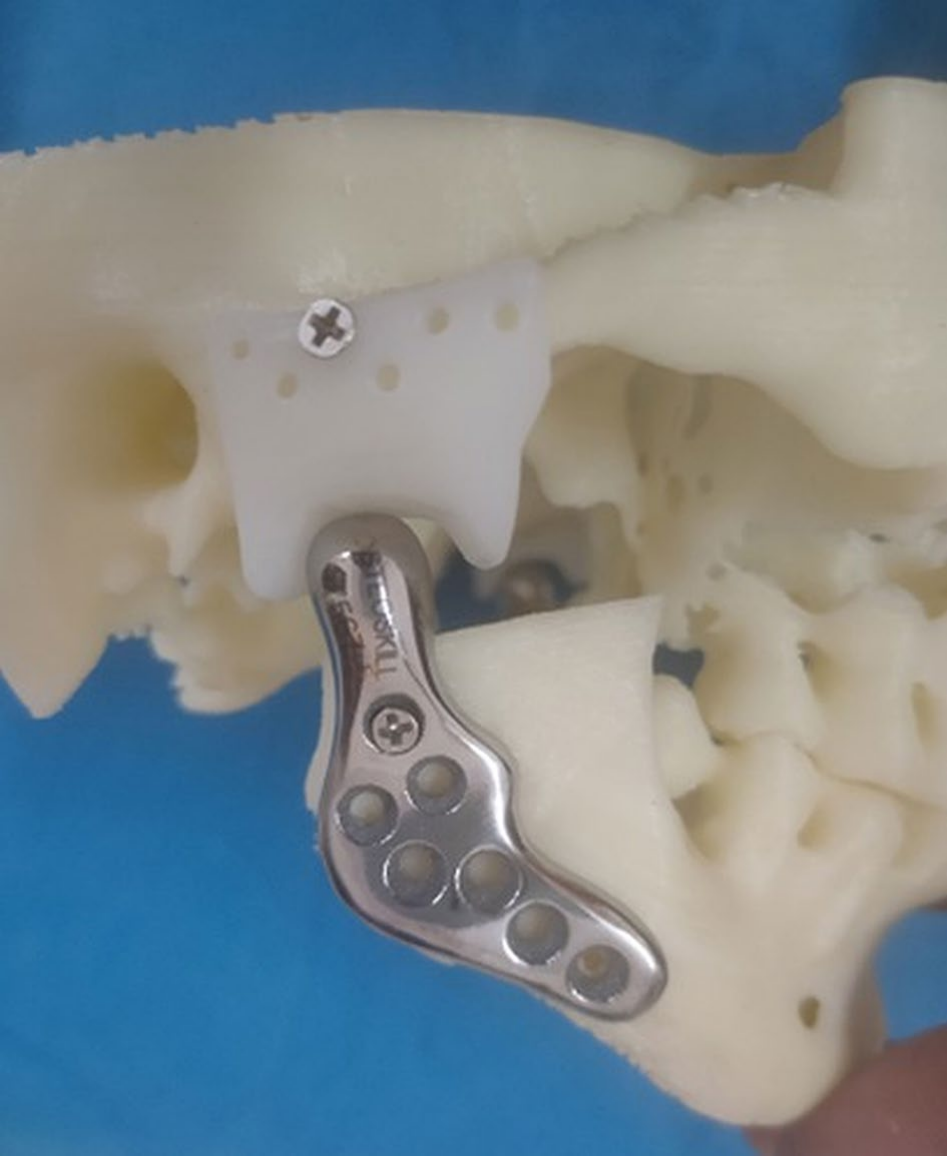
Mandible component with fossa on a 3D model.

In this study, indigenous custom-made 3 D printed titanium joint was used. The fossa was made up of ultra-high molecular weight polyethylene (Fig. 2).

Custom prosthesis was made up of titanium alloy grade 23 (Renishaw, UK, power) and prosthesis was manufactured using direct metal laser sintering (DMLS) (Renishaw, UK, power). The manufacturing unit has quality certification for patient specific implant manufacturing using titanium. ISO13485:2016.

#### Design process

The siemens computed tomography machine was used to obtain the DICOM data of the face of the patients included in the study. The DICOM data with slice thickness of 0.625 mm was then uploaded into the image processing software. In the present study slicer and blender software is used. Thresholding and segmentation of the desired anatomical region was done. Three-dimensional model was formulated and converted to a series of surface meshes and prepared for 3D printing through the addition of connectors and surface colour information. File was obtained in the standard triangle language (STL) format. This was further processed in 3D designing software. Blender software was used for implant designing.

#### Fabrication of implant

The build chamber was first filled with inert gas (argon) and heated to the optimal build temperature. A thin layer of metal powder was spread over the build platform and a high-power laser scanned the cross-section of the component, melting (or fusing) of the metal particles together was done and the next layer was formed. The entire area of the model was scanned and the part was built fully solid. When the scanning process was completed, the build platform moved down by one layer thickness and the recoater spreaded another thin layer of metal powder. The process is repeated until the whole part was completed.

When the build process was finished, the parts were fully encapsulated in the metal powder.

When the bin cools to room temperature, the excess powder was manually removed and the parts were typically heat treated while still attached to the build platform to relieve any residual stresses. Then the components were detached from the build plate via cutting or machining for the post processing process. After post-processing, the finished implant was cleaned using ultrasonic cleaning and then packed in Tyvek bag. Packaged implants get Gamma sterilised.

### Collection of specimens

The blood samples were collected from all the patients at 4 different timelines. The amount of blood sample withdrawn from each patient at single point is 3 ml. Patients were instructed to avoid use of cosmetics, carbonated drinks, food supplements and multivitamins. A total of 44 samples (4 each for 11 patients) were obtained and were stored at − 80 °C till the time of processing. The samples were kept outside the refrigerator for 2 h and allowed to attain room temperature before analysis.

### Metal ion analysis

Inductively coupled plasma mass spectrometry (ICP-MS) was used as an analytical method to detect metal ions in blood samples (Aligent technologies, 7800 ICP-MS, USA). Quantification of the metal ion levels was done by this machine based on their mass:charge ratio after generating metal ions and atoms by plasma. After analysing, the data obtained was formulated in tabular form.

### Data analysis

Data were analyzed by IBM SPSS version 21 (IBM Corp, Armonk, NY). The normality of data was checked by the Shapiro Wilk W test. Parametric tests were used to analyze the data. Paired t was used to compare various timelines. Mann Whitney test was used for comparison between two groups at different time intervals. A level of p < 0.05 was considered as statistically significant. Pearson correlation test was used to correlate the surface area of the joint with serum titanium ion levels.

### Ethical approval

This study was initiated after obtaining ethical clearance from the Institute Ethical Committee under the registration number: IECPG-698/19.12.2019.

## Results

The study sample comprised 11 patients (8 males, 3 females). The mean age of included patients is 24.73 ± 10.43. The number of unilateral cases was 6 and bilateral cases was 5. (Table 1) The mean surface of the TMJ TJR is 2724.14 square mm.

**Table 1:** Serum titanium ion levels of individual patients.

	Sex	Age	Side of ankylosis	Surface area of alloplastic joint in square mm	T0	T1	T2	T3
1	M	31	Bilateral	3532	0.1	25	34.33	14.4
2	M	23	Right	1722.35	0.87	5.6	8.5	32.49
3	M	14	Right	1585.64	0.23	38.92	40.29	41.759
4	M	20	Bilateral	3105.34	4.9	42.97	27.9	42.9
5	M	21	Bilateral	4596.4	3.7	9	14.65	43.22
6	F	19	Bilateral	2596.4	5.6	55.145	55.193	62.506
7	F	20	Right	1468.66	20.18	53.415	15.01	54.959
8	M	36	Left	2235.15	11.9	28	31	46
9	F	50	Bilateral	3244.33	22.7	17.04	44.525	63.375
10	M	17	Right	2345.51	21.45	62.37	17.46	63.931
11	M	21	Right	3533.79	11.145	58.182	59.641	61.551
Mean (SD)	M = 8 (72.7%) F = 3 (27.3%)	24.73 (10.43)	U/L = 6 (54.5%) B/L = 5 (45.5%)	2724.14	9.343 (8.70)	35.97 (20.27)	31.68 (17.03)	47.91 (15.47)
P value						0.009	0.032	0.000

Data is presented as mean (standard deviation) or as frequency.

*M* male, *F* female, *U/L* unilateral, *B/L* bilateral, *SD* standard deviation.

**Figure 3:**
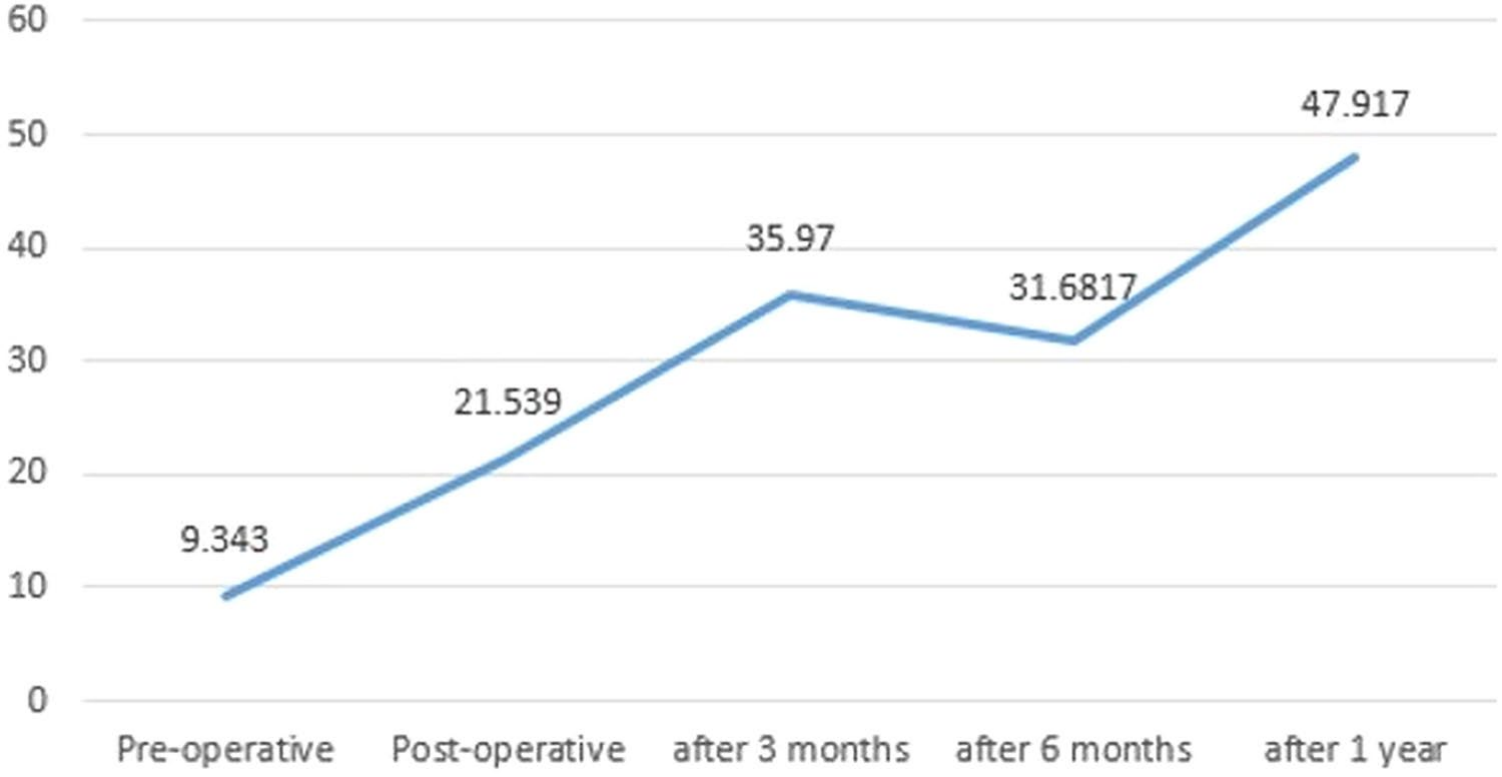
Linear graph showing serum titanium ion levels.

The mean serum titanium ion levels at T0, T1, T2, and T3 were 9.34 ± 8.70 mcg/L, 35.97 ± 20.27 mcg/L, 31.68 ± 17.03 mcg/L, and 47.91 ± 15.47 mcg/L respectively (Table 1, Fig. 3). The mean serum titanium ion levels increased significantly at T1 (p = 0.009), T2 (p = 0.032), and T3 (p = 0.00) interval. Intergroup comparison between unilateral and bilateral groups was done (Table 2). In unilateral cases, mean serum titanium ion levels at T0, T1, T2, and T3 were 10.96 ± 9.08 mcg/L, 53.22 ± 10.21 mcg/L, 28.18 ± 21.28 mcg/L, and 50.93 ± 13.43 mcg/L. In bilateral cases, mean serum titanium ion levels at T0, T1, T2, and T3 were 7.40 ± 8.81 mcg/L, 31.03 ± 21.64 mcg/L, 35.31 ± 15.51 mcg/L, and 45.28 ± 19.92 mcg/L. No significant difference was observed in serum titanium levels between the unilateral and bilateral groups. The correlation of the surface area of the TMJ TJR with serum titanium ion levels is mentioned in Table 3. No significant difference was observed. None of the patients showed any signs and symptoms of metallosis like pain, fever, tenderness, crepitus, loosening of the prosthesis, skin discoloration around the prosthesis, and systemic complications.

**Table 2:** Serum titanium ion levels in unilateral and bilateral groups.

	Mean titanium levels (SD)		p-value
	Unilateral	Bilateral	
Pre-operative	10.96 (9.08)	7.40 (8.81)	0.528
Post-operative	18.13 (15.20)	25.63 (17.43)	0.465
After 3 months	53.22 (10.21)	31.03 (21.64)	0.113
After 6 months	28.18 (21.28)	35.31 (15.51)	0.561
After 1 year	50.93 (13.43)	45.28 (19.92)	0.613

Data is presented as mean (standard deviation).

**Table 3:** correlation of surface area with metal ion levels.

Time interval	Unilateral	Bilateral
3 months	0.407	0.068
6 months	0.109	0.055
12 months	0.226	0.528

Data is presented as p value (< 0.05 is considered as statistically significant).

## Discussion

Total temporomandibular joint replacement (TMJ TJR) is a successful surgical treatment for irreparably damaged, ankylosed joints, end-stage temporomandibular joint disorders and is associated with high rates of patient satisfaction and improvement in quality of life and overall physical function^17^. In an ideal scenario, prostheses made up of biocompatible materials should not wear with either function or time, yet there is increasing recognition that the metal debris of the prosthetic material may be associated with untoward local tissue reactions and systemic complications^10,18^. These complications are well researched and described in the details in the orthopedic literature^18^.

Various methods have also been described for early diagnosis of metallosis in the literature including analysis of synovial fluid, serum, whole blood, and radiographic interventions. With time and advancing technology, what happens to implant inside the body has become the question of interest. Here comes the role of tribocorrosion which can affect any implant that has been placed in the human body or inside other chemical solution leading to the erosion of metal surface thereby affecting its performance^19^. Many studies have been designed documenting the tirbocorrosion behavior of metallic implants. In vitro studies have also been done explaining the tribocorrosive behavior in TMJ TJR devices^20^. Inspired from the orthopedic literature, Mercuri et al. did studies on failed, retrieved TMJ TJR and summarized the role of wear and corrosion interactions on the failure of TMJ TJR^8^. Abundant literature is also available on dental implants documenting the metal ion levels in saliva and its effect on implant life^16,21^. However, only one cross sectional study regarding the serum metal ion levels was reported in the literature for TMJ TJR patients^16^. No longitudinal study regarding serum metal levels in TMJ TJR patients exists in the English literature, as per our knowledge.

Even though Ti6AlV4 has been the major alloy used in implantable metal devices, concern over reports of the alloy's aluminum and/or vanadium content leading to hypersensitivity has led to investigation of novel titanium alloys with similar or improved mechanical and clinical properties by incorporation of non-toxic alloying elements such as niobium, molybdenum, tantalum, zirconium and tin in the formulation of the alloy^22–24^. So called beta titanium alloys (e.g. TiZrMoFe) also have the theoretical advantage of a lower elastic modulus and therefore lower stress-shielding at implant-bone interfaces. However, there are increasing reports of excessive wear of femoral stems made of such alloys compared to the Ti6Al4V gold standard^25–28^. These wear particles of titanium ions were associated with inflammatory reactions, cytotoxic reactions, lung problems, fibrosis, and tumors. The syndrome associated with titanium ions is yellow nail syndrome which is characterized by bronchial obstruction, lymphedema, and a yellow color change in nails, pleural effusion, bronchiectasis, and maxillary sinus inflammation^29,30^.

The major reason to use the 3D printed prosthesis in this study was the compromised fit and adaptability of the smallest available stock joint in Indian population^31^. This custom design also has an advantage of the improved fit which provides increased stability to the joint and result in reduced wear in the long run associated with the debris of polyethylene as seen in alloplastic knee and hip prosthesis^32,33^.

Various methods have been reported to diagnose tribocorrosion at an early stage including serum metal ion analysis, biosensors to detect metal ions in body fluids, joint fluid analysis. The most used technique is inductive coupled plasma- mass spectrometry (ICPMS). The whole blood level of metal ions usually indicates the cumulative effect^34^ and the serum level of metal ions indicates the recent effect of the wear and tear process^35^. Thus in this study, serum metal ion levels were used.

In this longitudinal study, at T0, the mean serum level of titanium observed were higher than the reference range set by mayo laboratories (< than 1 ng/L)^36^. However actual numerical values cannot be compared because of differences in metallurgy, methods of analysis, and measurement units. The reasons for raised values may be attributed to dietary supplements, cosmetics, environment, water contamination due to industrial wastes, and occupation^37^. This preoperative sample gives the study a good strength and also eliminates the possibility of confounding factors.

At T1 and T2, there is a significant rise in serum titanium levels (p = 0.009) and (p = 0.032) respectively. All the patients included in the present study had ankylosis of the TMJ for which TMJ TJR was done. According to the study done by Kaban et al.^38^ Roychoudhury et al.^39^ Mercuri et al.^40^ aggressive physiotherapy plays a crucial role in preventing the heterotopic bone formation around the joint in the early postoperative period. The most critical period in which the highest quantity of heterotopic bone is formed is during the first 3 months after surgery^41^. The physiotherapy with acrylic Archimedean screw was judiciously followed for 6 months to stabilize the adequate mouth opening^42^. This aggressive physiotherapy and the masticatory load may be the reason for the raised titanium ions levels at 3 months and 6 months interval in surrounding tissues and ultimately going to the systemic circulation^43^. The above is just notional theorization as the TMJ TJR prosthesis design was not metal to metal but metal to UHMWPE.

At T3, increase in serum titanium ion levels is found to be statistically significant (p = 0.00). The reason may be the continuous cyclic loading on the prosthesis due to normal functioning. Although the bearing surface contact is more in bilateral prosthesis when compared to a unilateral group and the observed values are more in the bilateral group, but the difference is not statistically significant. Similar results have also been documented in the literature^44,45^.

The manufacturing of the prosthesis, the type of alloy used has an impact on the corrosive demeanor of the prosthesis. In the orthopedic literature, different metal composition of head and neck junction results in more serum metal ion release. The angle of the femoral head in cognation to neck also has a role in the release of serum cobalt, chromium, and titanium ions^46^. However, this categorical design is not noted with respect to the temporomandibular joint prosthesis.

When the time trend over 1 year was observed, the increased serum titanium ions which is 2–3 times more than the preoperative period may be due to the initial wear phase of the prosthesis^47^. As per the work done by Tipper et al.^47^ there are two phases of wear in a life of prosthesis- initial wear during the first year of life of the prosthesis in function, after which there is steady-state wear. The second phase of wear commences when the prosthesis becomes loose and there is a rise in serum metal ions levels. The initial wear phase may denote the running-in period of the prosthesis which states that the simulatory effect of cyclic load on the prosthesis causes the release of metal ions as a result of abrasion^48^. The second phase of wear suggests corrosive erosion of the prosthesis^48^.

In the present study, at each follow-up level, none of the patients had serum titanium ion levels that reach the reported high calibers of metal ions as mentioned in studies done by Bradberry et al.^3^ and all the patients enrolled in the study are having fully functional prosthesis in situ without any signs and symptoms.

Results of our study suggest that there is a positive correlation of the running-in period of TMJ TJR on the serum metal ion levels that is consistent with the results of a study done by Heisel et al. after hip metal resurfacing arthroplasty^48^.

Recently Onoriobe et al.^49^ did a study in the United States population and concluded that there will be a 58% ascend in demand for total temporomandibular joint replacements by the year 2030. The present study will give an idea as to what is happening with metal ion release in these joints after being implanted into the patients. The authors will follow the patients yearly for a long period of time to look for any signs and symptoms of metallosis. Longitudinal study design like ours has the advantage of exploring patterns of change and dynamics of titanium ion levels with time, but the constraints include a small number of analyzed subjects and a short follow-up. The effect of potable water on the serum ion levels have also not been studied on the patients. The preoperative serum ion levels however act as control. Disadvantage of this study is small sample size and less follow up. Ours is a preliminary study that was designed to know the trend of serum titanium ion levels in 3D printed TMJ TJR patients with time and whether increased serum titanium levels are associated with any signs and symptoms. Further research with large sample size and longer follow-ups are recommended.

## Conclusion

This preliminary research concluded that serum titanium ion levels increased after TMJ TJR. Serum titanium ion continued to show increased levels till the last follow-up of 1 year. These initial increase in serum titanium ion levels is due to the initial wear phase of the prosthesis which manifests over 1 year. Though the Ti ions are leaching in systemic circulation but are not causing any untoward systemic or local complications. Further studies with large sample sizes and long-term follow-up are required to see the deleterious effect if any, of patient specific TMJ TJR on human body.

## Data Availability

The raw/processed data required to reproduce these findings are shared in Table 1.

## References

[1] Mercuri LG, Mercuri LG (2016). History of TMJ TJR. Temporomandibular Joint Total Joint Replacement–TMJ TJR.

[2] Yadav P, Roychoudhury A, Kumar RD, Bhutia O, Bhutia T, Aggarwal B (2021). Total alloplastic temporomandibular joint replacement. J. Maxillofac. Oral Surg..

[3] Bradberry SM, Wilkinson JM, Ferner RE (2014). Systemic toxicity related to metal hip prostheses. Clin. Toxicol..

[4] Prasad K, Bazaka O, Chua M (2017). Metallic biomaterials: Current challenges and opportunities. Materials (Basel).

[5] Heffernan EJ, Alkubaidan FO, Nielsen TO, Munk PL (2007). The imaging appearances of metallosis. Skelet. Radiol..

[6] Miki H, Sugano N, Yamamura M, Nakamura N, Nishii T, Yoshikawa H (2006). Serious metallosis of a metal head due to fragmented ceramic screws in a cemented THA. Arch Orthop. Trauma Surg..

[7] Mathew MT, Kerwell S, Lundberg HJ, Sukotjo C, Mercuri LG (2014). Tribocorrosion and oral and maxillofacial surgical devices. Br. J. Oral Maxillofac. Surg..

[8] Mercuri LG, Mathew MT, Kerwell S, Lundberg H, Sukotjo C (2015). Temporomandibular joint replacement device research wear and corrosion technology transfer from orthopedics. J. Bio-and Tribo-Corrosion.

[9] Ude CC, Esdaille CJ, Ogueri KS (2021). The mechanism of metallosis after total hip arthroplasty. Regen Eng. Transl. Med..

[10] Bijukumar DR, Segu A, Souza JCM (2018). Systemic and local toxicity of metal debris released from hip prostheses: A review of experimental approaches. Nanomedicine.

[11] Mercuri LG, Urban RM, Hall DJ, Mathew MT (2017). Adverse local tissue responses to failed temporomandibular joint implants. J. Oral Maxillofac. Surg..

[12] Marinescu R, Socoliuc C, Botezatu I, Laptoiu D, Voinescu D (2017). Metallosis-literature review and particular cases presentation. Key Eng. Mater..

[13] Neto MQ, Radice S, Hall DJ, Mathew MT, Mercuri LG, Pourzal R (2022). Alloys used in different temporomandibular joint reconstruction replacement prostheses exhibit variable microstructures and electrochemical properties. J. Oral Maxillofac. Surg..

[14] Chang JD, Lee SS, Hur M, Seo EM, Chung YK, Lee CJ (2005). Revision total hip arthroplasty in hip joints with metallosis: A single-center experience with 31 cases. J. Arthroplasty.

[15] Ollivere B, Darrah C, Barker T, Nolan J, Porteous MJ (2009). Early clinical failure of the Birmingham metal-on-metal hip resurfacing is associated with metallosis and soft-tissue necrosis. J. Bone Joint Surg. Br..

[16] Mercuri LG, Miloro M, Skipor AK, Bijukumar D, Sukotjo C, Mathew MT (2018). Serum metal levels in maxillofacial reconstructive surgery patients: A pilot study. J. Oral Maxillofac. Surg..

[17] Wolford LM, Mercuri LG, Schneiderman ED, Movahed R, Allen W (2015). Twenty-year follow-up study on a patient-fitted temporomandibular joint prosthesis: The Techmedica/TMJ Concepts device. J. Oral Maxillofac. Surg..

[18] Diomidis N, Mischler S, More NS, Roy M (2012). Tribo-electrochemical characterization of metallic biomaterials for total joint replacement. Acta Biomater..

[19] Mathew MT, SrinivasaPai P, Pourzal R, Fischer A, Wimmer MA (2009). Significance of tribocorrosion in biomedical applications: Overview and current status. Adv. Tribol..

[20] Royhman D, Yuan JC, Shokuhfar T, Takoudis C, Sukotjo C, Mathew MT (2013). Tribocorrosive behaviour of commonly used temporomandibular implants in a synovial fluid-like environment: Ti–6Al–4V and CoCrMo. J. Phys. D Appl. Phys..

[21] Gopi G, Shanmugasundaram S, Krishnakumar Raja VB, Afradh KM (2021). Evaluation of serum metal ion levels in dental implant patients: A prospective study. Ann. Maxillofac. Surg..

[22] De Meurechy N, Mommaerts MY (2018). Alloplastic temporomandibular joint replacement systems: A systematic review of their history. Int. J. Oral Maxillofac. Surg..

[23] Siemers C, Bäker M, Brunke F, Wolter D, Sibum H, Froes FH, Qian MBTT (2018). 46—Aluminum- and vanadium-free titanium alloys for application in medical engineering. Woodhead Publishing Series in Biomaterials.

[24] Mercuri L (2020). Alloplastic temporomandibular joint replacement—What does the future hold?. Front. Oral Maxillofac. Med..

[25] Çaha I, Alves AC, Chirico C (2022). Tribocorrosion-resistant Ti40Nb-TiN composites having TiO(2)-based nanotubular surfaces. ACS Biomater. Sci. Eng..

[26] Wang K (1996). The use of titanium for medical applications in the USA. Mater. Sci. Eng. A.

[27] Morlock MM, Dickinson EC, Günther KP, Bünte D, Polster V (2018). Head taper corrosion causing head bottoming out and consecutive gross stem taper failure in total hip arthroplasty. J. Arthroplasty.

[28] Yang X, Hutchinson CR (2016). Corrosion-wear of β-Ti alloy TMZF (Ti-12Mo-6Zr-2Fe) in simulated body fluid. Acta Biomater..

[29] Emerson PA (1966). Yellow nails, lymphoedema, and pleural effusions. Thorax.

[30] Zefras AJ (1966). Yellow nail syndrome with bilateral bronchiectasis. Proc. R. Soc. Med..

[31] Alagarsamy R, Roychoudhury A, Bhutia O, Lal B, Yadav R, Bhalla AS (2021). Evaluation of fit feasibility of stock total joint replacement in temporomandibular joint ankylosis patients. Br. J. Oral Maxillofac. Surg..

[32] Gruber EA, McCullough J, Sidebottom AJ (2015). Medium-term outcomes and complications after total replacement of the temporomandibular joint. Prospective outcome analysis after 3 and 5 years. Br. J. Oral Maxillofac. Surg..

[33] Mercuri LG (2012). Alloplastic temporomandibular joint replacement: Rationale for the use of custom devices. Int. J. Oral Maxillofac. Surg..

[34] Paustenbach DJ, Galbraith DA, Finley BL (2014). Interpreting cobalt blood concentrations in hip implant patients. Clin. Toxicol. (Phila).

[35] Simonsen LO, Harbak H, Bennekou P (2012). Cobalt metabolism and toxicology–a brief update. Sci. Total Environ..

[36] Yao JJ, Lewallen EA, Thaler R (2020). Challenges in the measurement and interpretation of serum titanium concentrations. Biol. Trace Elem. Res..

[37] Kovochich M, Finley BL, Novick R (2018). Understanding outcomes and toxicological aspects of second generation metal-on-metal hip implants: A state-of-the-art review. Crit. Rev. Toxicol..

[38] Kaban LB, Bouchard C, Troulis MJ (2009). A protocol for management of temporomandibular joint ankylosis in children. J. Oral Maxillofac. Surg..

[39] Yadav P, Roychoudhury A, Bhutia O (2021). Strategies to reduce re-ankylosis in temporomandibular joint ankylosis patients. Br. J. Oral Maxillofac. Surg..

[40] Mercuri LG, Saltzman BM (2017). Acquired heterotopic ossification of the temporomandibular joint. Int. J. Oral Maxillofac. Surg..

[41] Matta JM, Siebenrock KA (1997). Does indomethacin reduce heterotopic bone formation after operations for acetabular fractures? A prospective randomised study. J. Bone Joint Surg. Br..

[42] Roychoudhury A, Parkash H, Trikha A (1999). Functional restoration by gap arthroplasty in temporomandibular joint ankylosis: A report of 50 cases. Oral Surg. Oral Med. Oral Pathol. Oral Radiol. Endod..

[43] Sauvé P, Mountney J, Khan T, De Beer J, Higgins B, Grover M (2007). Metal ion levels after metal-on-metal Ring total hip replacement: A 30-year follow-up study. J. Bone Joint Surg. Br..

[44] Luetzner J, Krummenauer F, Lengel AM, Ziegler J, Witzleb WC. Serum Metal Ion Exposure after Total Knee Arthroplasty. *Clin Orthop Relat Res*. 2007;461. https://journals.lww.com/clinorthop/Fulltext/2007/08000/Serum_Metal_Ion_Exposure_after_Total_Knee.28.aspx. 10.1097/BLO.0b013e31806450ef 17438467

[45] Van Der Straeten C, Banica T, De Smet A, Van Onsem S, Sys G (2017). Metal ion measurements from total knee arthroplasty. Orthop. Proc..

[46] Pourzal R, Lundberg HJ, Hall DJ, Jacobs JJ (2018). What factors drive taper corrosion?. J. Arthroplasty.

[47] Tipper JL, Ingham E, Jin ZM, Fisher J (2005). (iv) The science of metal-on-metal articulation. Curr. Orthop..

[48] Heisel C, Streich N, Krachler M, Jakubowitz E, Kretzer JP (2008). Characterization of the running-in period in total hip resurfacing arthroplasty: An in vivo and in vitro metal ion analysis. J. Bone Joint Surg. Am..

[49] Onoriobe U, Miloro M, Sukotjo C, Mercuri LG, Lotesto A, Eke R (2016). How many temporomandibular joint total joint alloplastic implants will be placed in the United States in 2030?. J. Oral Maxillofac. Surg..

